# Comprehensive Physiotherapy Rehabilitation in a 25-Year-Old Female With Nonspecific Low Back Pain: A Case Report

**DOI:** 10.7759/cureus.60514

**Published:** 2024-05-17

**Authors:** Nikita Kaple, Pratik Phansopkar

**Affiliations:** 1 Musculoskeletal Physiotherapy, Ravi Nair Physiotherapy College, Datta Meghe Institute of Higher Education and Research, Wardha, IND

**Keywords:** william's flexion exercise, modified oswestry disability questionnaire, modality, physiotherapy, low back pain

## Abstract

A prevalent musculoskeletal disorder known as nonspecific low back pain (NSLBP) is characterized by lumbar discomfort or pain that lacks a distinct, identifiable etiology. It is the main root of disability in all corners of the globe, affecting individuals across diverse age groups and occupations. NSLBP is often categorized as a multifactorial condition, encompassing a range of potential contributing factors such as poor posture, sedentary lifestyle, muscle imbalances, and psychosocial elements. According to current standards, there is a good prognosis for acute nonspecific back pain, although this prognosis is mostly reliant on return to function. Various treatment strategies are available, totally reliant upon the underlying cause of the discomfort. This case report presents the combination of traditional therapy and William's flexion exercises in a 25-year-old female nursing student who presented with complaints of low back pain (LBP) for the last three months. This study investigates the effect of William's flexion exercises in nonspecific low back pain to manage pain and range of motion (ROM), and improve the overall quality of life, which was evaluated using the visual analog scale (VAS), modified Schober's test, pressure biofeedback unit, and modified Oswestry disability questionnaire. The patient received an enhanced physiotherapy program that increased the flexibility and range of motion in the lumbar extensor, hip flexor, and hamstring muscles. The outcome measure shows notable gains after the therapeutic interventions.

## Introduction

One of the most common muscular ailments is low back pain (LBP) associated with the workplace; it is a significant contributor to disability among physiotherapists. The lower back is made up of the lumbar and sacral regions, and at least once a year, 50% of people have low back pain [1]. Up to 90% of people are expected to have lower back pain at some point in their lives, as it is the main factor leading to impairment and inability to work. Any individual may have lower back pain; it is a persistent multi-etiological ailment that can be considered a lumbar spine illness [2]. Low back pain is the most common cause of limitation of activity and absence in general, which has a lifetime prevalence of up to 83% and a point prevalence of 19%-39%. Nonspecific low back pain (NSLBP) makes up over 85% of cases and is defined as LBP without a particular disease, such as a tumor, fracture, or inflammation [3]. Nonspecific low back pain is a major worldwide health ailment that affects people of all ages. According to management recommendations, triage should be performed to identify the small number of low back pain cases that are brought on by really significant medical conditions and necessitate referral to a specialist, a diagnostic workup, or both. As the reason for generalized low back pain is unknown from a pathoanatomical perspective, treatment aims to reduce discomfort and its side effects [4]. Of all instances of LBP, 90% are vague and affect people of various ages. LBP has an impact on quite a portion of the general population, among athletes, students, and the elderly population.

Despite being quite frequent, low back discomfort is "nonspecific" since many individuals' underlying reasons for the pain are uncertain. Muscular strength and spine stability are important variables in the development and maintenance of nonspecific LBP. The great majority of patients indicate nonspecific LBP, which is defined as pain from the gluteal fold to the upper lumbar vertebrae of unclear etiology [5]. The absence of structural changes that might cause back pain, including shortened disc space, compression of the nerve roots, fractures of the bones or joints, and severe lordosis or scoliosis, emphasizes NSLBP. One of the most common causes of missed work globally is nonspecific low back discomfort even when there is no structural change in the condition. It can hinder everyday activities and result in a temporary or permanent incapacity to work [6]. Point discomfort and paraspinal muscular spasms are frequent causes [7]. Essentially, less than 5% of all back issues in the general adult population are caused by more serious etiologies, with muscle strains and ligament sprains accounting for up to 97% of back pain consequences [5]. Workers who perform hard physical labor, such as lifting weights, performing repetitive motions, or maintaining static postures regularly, have a greater prevalence of nonspecific low back pain. Several risk factors are linked to low back pain (LBP), including physical aspects of hard lifting, bending over, and twisting [8].

The primary intervention for conservative treatment is physiotherapy, which employs a variety of modalities and therapeutic techniques to restore function and strengthen and stabilize the spine. Physical treatment can help alleviate low back pain symptoms and increase or restore mobility, allowing for the return of normal function. According to research, the first line of treatment consists of steroid injections, exercise, medicine for pain management, and physical therapy; however, the proposed treatments lack any particular guidelines or standards for physical therapy treatment. To assess the efficacy of a physical therapy rehabilitation plan for a female patient who had been referred for nonspecific low back pain, this case study was conducted. To improve the patient's quality of life, this study combined William's flexion exercises, postural control exercises, upper and lower limb strengthening activities, aerobic training, and traditional physiotherapy [9].

## Case presentation

**Figure 1:**
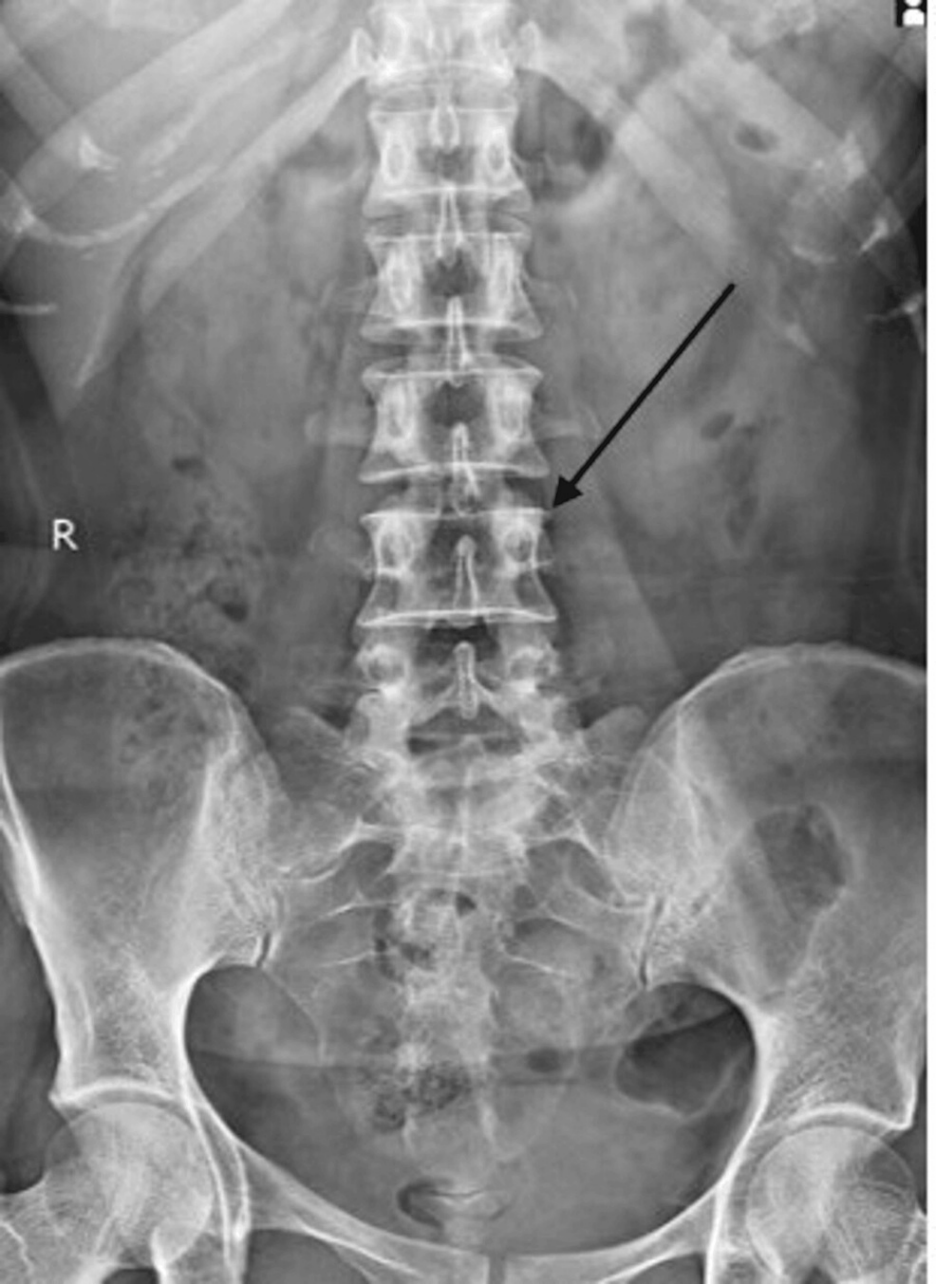
X-ray of the lumbosacral spine showing vertebral bodies with normal density Pedicle and spinous processes are intact. The sacroiliac joint appears normal. There is no demonstrated pathology.

A 25-year-old female nursing student with a height of 154 cm, weight of 60 kg, and body mass index of 25.3 kg/m^2^ presented with chief complaints of lower back pain for three months. She gave a history of lifting heavy objects three months back. The pain was gradually progressive. The visual analog scale (VAS) indicated that the pain was 4 out of 10 during rest and 7 out of 10 during activity. The patient's pain was aggravated due to prolonged sitting, standing, forward bending, and performing high-intensity activities such as lifting heavy weights during activities of daily living and relieved on rest. She had difficulty in maintaining postures for prolonged periods. The patient also visited the orthopedic department with the same complaint where physical examination and investigations were done. X-ray reveals no abnormal finding as shown in Figure 1. Physical examination suggests a paraspinal spasm for which she was advised to take medication. After seven days of treatment, she was not relieved, so she was advised to take physiotherapy treatment.

Clinical finding

Before the examination, the patient's and her family member's consent was obtained. The patient was examined in the prone lying position. Bilateral paraspinal spasms were present in the lower back area. Grade 2 tenderness was present on bilateral paraspinal muscles in the L3-L4 region. There was no radiating pain to the lower limb, which was confirmed by the slump test indicating no neural involvement. A significant reduction in the lumbar spine's range of motion (ROM) occurred and was noted in the outcome section. The range of motion of the hip joint was painless and incomplete. The results of the modified Schober's test indicate a considerable reduction of lumbar flexion, with a difference of 1.5 cm. The lumbar extensor and flexor strength were reduced. The lumbar flexors showed a grade of 3+, whereas the lumbar extensors showed a grade 3 muscle strength on manual muscle testing. Using the pressure biofeedback, the core strength was evaluated, which displays the 85 mmHg deflection. Functional limitations or disabilities were evaluated using the modified Oswestry disability scale. The patient had a modified Oswestry disability index score of 46%, indicating a severe disability that hampered her activities of daily living. Table 1 presents the timeline for this case report.

**Table 1:** Timeline of events OPD: outpatient department, LBP: low back pain

Date	Events
16/11/2023	The patient came to orthopedic OPD with a complaint of low back pain.
17/11/2023	A radiological investigation was done, but no finding was found. On clinical presentation and diagnostic findings, a diagnosis of nonspecific LBP was made. The patient was referred to physiotherapy.
18/11/2023	A physiotherapy rehabilitation session was started.
2/12/2023	Physiotherapy intervention ended, and the patient was advised to adhere to the home program.

Physiotherapy rehabilitation

Following the examination, the patient was instructed to receive physical therapy sessions for four weeks. The treatment plan will include patient education, pain management strategies, a range of motion and strength training plan, posture training, and a home exercise program. Furthermore, the intervention will focus on balance and proprioceptive training. Table 2 shows the rehabilitation of low back pain.

**Table 2:** Rehabilitation of low back pain

Goals	Therapeutic intervention	Rationale
Patient education for the condition and physiotherapy intervention	Patient education plays a crucial role in the treatment process by giving patients the knowledge and self-assurance that they need to comprehend their condition, take an active role in their rehabilitation, and effectively maintain their health. Ensuring the patient how the treatment works to regain activities of daily living.	Advice on managing the disease, taking an active role in the rehabilitation
To alleviate low back pain and enhance patient's well-being	Thermotherapy: a hydrocollator pack was given for 20 minutes. The temperature of the water was 170 degrees Fahrenheit (76.7 degrees Celsius). A minimum of six layers of towel was used between the pack and the skin.	To lessen the patient's low back pain symptoms and impairment
	Interferential therapy was given to the patient in the prone position on the lumbar region to reduce pain and muscle spasms, with a beat frequency of 50 Hz for 10 minutes with four electrodes in a vector pattern (5 × 9 cm).	To reduce pain and muscle spasms
To improve range of motion and strength of the lumbar spine	William's flexion exercises were given: gentle stretching exercises such as pelvic tilt, hamstring and hip flexor stretches, half sit-ups, single and double knee-to-chest exercises, and squats. Each exercise included a 10-second hold, 30 repetitions × 3 sets for 30-40 minutes on one side and then the same for the other side. These exercises were done gently and within a pain-free range of motion to prevent any potential strain or injury.	To help preserve flexibility and enhance the lumbar trunk and abdominal muscles' strength and endurance
To enhance the muscular strength of abdominal muscles and improve the patient's endurance level	Core-strengthening exercises help to build strength and endurance of abdominal muscles, which significantly reduces pain and improves functionality in the lower back. Exercises such as pelvic tilt, cat-cow position, bird dog, high and low planks, crunches, and Swiss ball (or exercise ball) exercises are effective in strengthening the core and relieving back discomfort.	To improve the strength and stability of the muscles, which have been weakened due to lower back pain
To improve balance and stability	Balance training activities may be used to improve proprioception. Proprioceptive tasks, such as single-leg balance and wobble board exercises, were included. Plyometric exercises were given to improve dynamic stability. Exercise complexity and difficulty can be increased gradually.	To enhance physical functioning, less fear of falling, faster walking, and improve fall-related self-efficacy
To improve postural alignment advice for lifestyle modification and ergonomics advice	Avoid soft and squashy seats. The patient was advised to use lumbar rolls to support the lower back while sitting or driving. Advice to use ergonomic chairs for work or long-term sitting. When lifting heavy weights, the patient bends through the knees as we do squats while maintaining the back straight. Avoid clear waist bending. The patient is advised to sleep on the side or back, as these postures support the preservation of spinal alignment, not to sleep on the stomach.	To correct postural habits and use appropriate infrastructure at work to avoid and cure low back pain

Home-based exercise program

**Figure 2:**
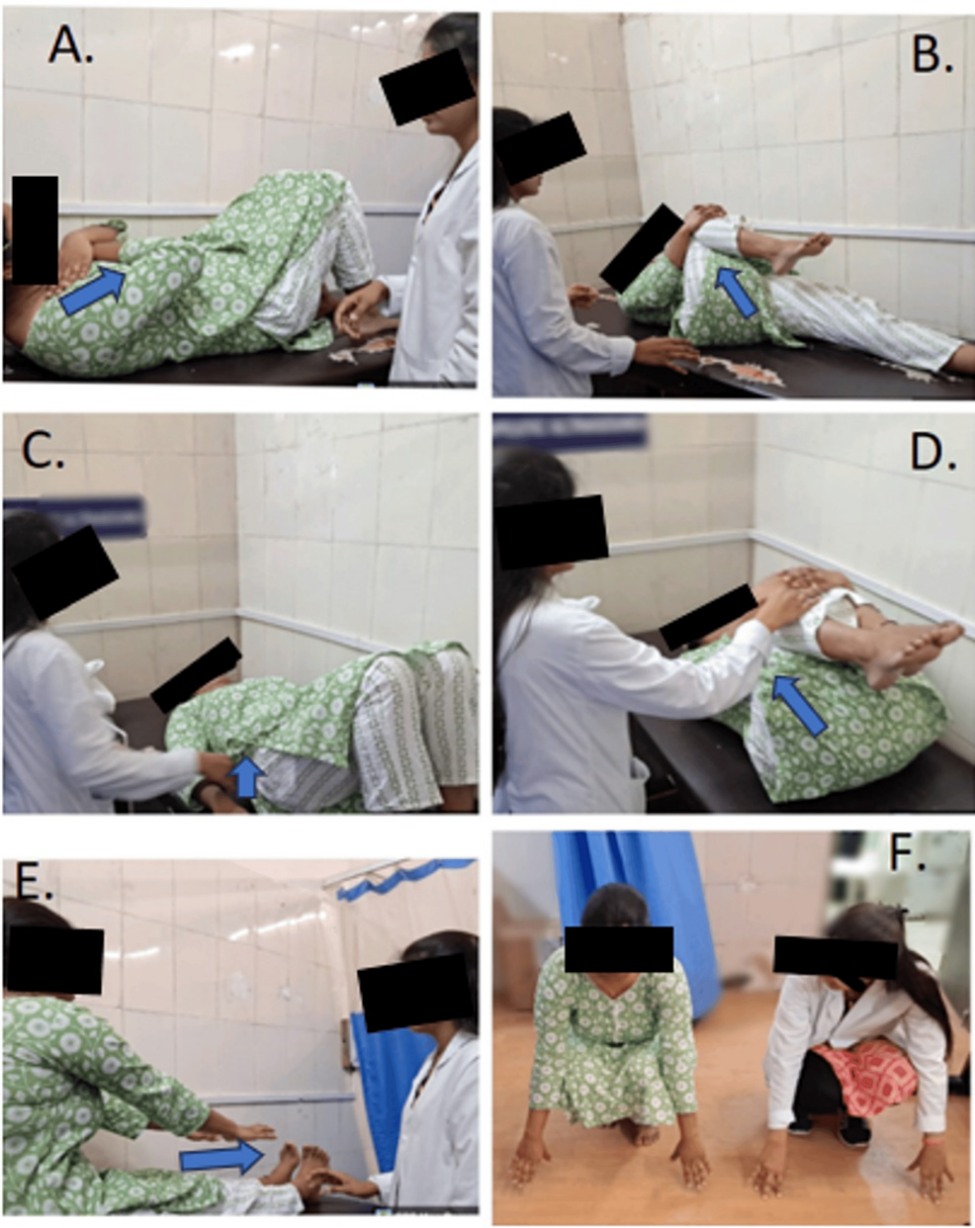
Patient performing William's flexion exercises A: The patient performing sit-ups in knee flexion. B: The patient performing a single knee-to-chest exercise to stretch the erector spinae. C: The patient performing a pelvic tilt. D: The patient performing a double knee-to-chest exercise to stretch the erector spinae. E: The patient performing seated reach to toes to stretch the hamstring and erector spinae. F: The patient performing squats.

To maintain proper posture while working and unwinding, the patient was advised to adhere to the exercise plan and ergonomic recommendations. Even when a particular sitting posture looks comfortable, it is important to change the posture twice hourly. Strength-based exercise done as part of daily activities has been shown to significantly reduce pain intensity and functional limitation. Exercises such as hip flexor stretching, hamstring stretching, thoracolumbar fascia strengthening, McGill curl-up exercise, and bird dog exercise were advised to be performed at home with 10 repetitions of one set with 30-second holds. Figure 2 shows William's flexion exercises.

Outcome measures

**Figure 3:**
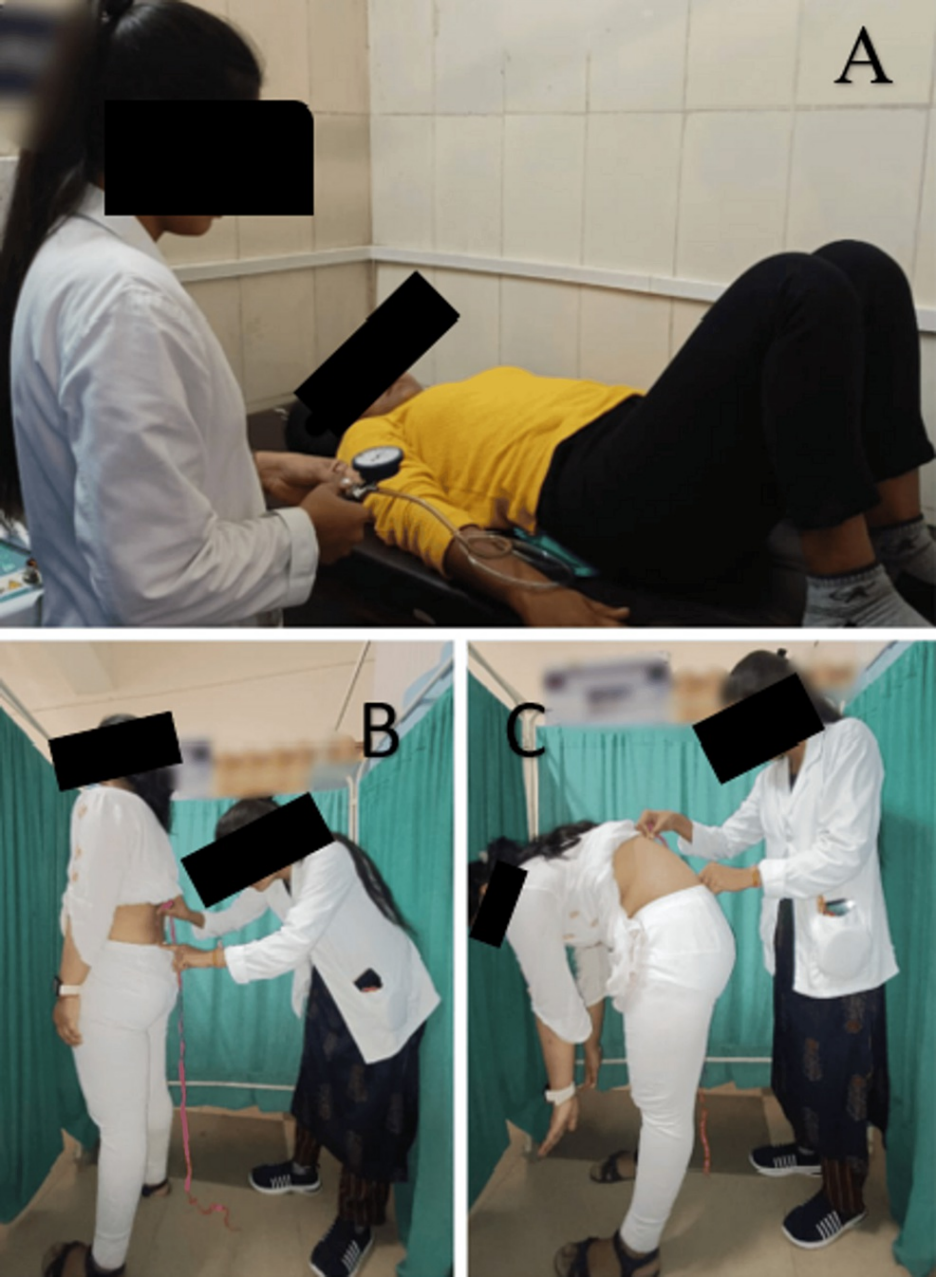
Core muscle strength measured via a pressure biofeedback unit (A) and assessment of the modified Schober's test for lumbar flexion (B and C)

Following four weeks of therapy, the patient underwent evaluation and showed improvement. Table 3 shows the outcome measures. Figure 3A shows the core muscle strength measured via a pressure biofeedback unit. Table 3 also shows the assessment of the modified Schober's test for lumbar flexion.

**Table 3:** Outcome measures VAS: visual analog scale, PBU: pressure biofeedback unit, MODQ: modified Oswestry disability questionnaire

Outcome measure	Pretreatment value	Post-treatment values after 4 weeks
VAS at rest	4.2 cm out of 10 cm	1.5 cm out of 10 cm
VAS at activity	7.3 cm out of 10 cm	2.2 cm out of 10cm
Modified Schober's test for lumbar flexion	Shows a difference of 1.5 cm	Shows a difference of 5.7 cm
Manual muscle testing for lumbar flexor	Grade 3+ (hold test position against slight pressure)	Grade 4 (hold test position against moderate pressure)
Manual muscle testing for lumbar extensor	Grade 3 (hold test position no added pressure)	Grade 4 (hold test position against moderate pressure)
PBU to check the strength of the core muscle	40 mmHg	135 mmHg
MODQ	23 out of 50 (46%) (severe disability)	4 out 50 (8%) (minimal disability)

## Discussion

This case report represents a case of a 25-year-old female with nonspecific LBP. The main purpose of William's flexion exercises was to reduce pain, increase ROM and flexibility, increase muscle strength, decrease disability, and increase quality of life. Core-strengthening exercises help to build strength and endurance of abdominal muscles, which significantly reduces pain and improves functionality in the lower back, improving the strength and stability of the muscles that have been weakened due to lower back pain. Balance training activities may be used to improve proprioception. Proprioceptive tasks, such as single-leg balance and wobble board exercises, correct postural habits. Appropriate infrastructure at work should be used to avoid and cure low back pain. The patient was advised to use lumbar rolls to support the lower back while sitting or driving. William's flexion exercise regimen attempts to lessen discomfort or soreness, offer lower trunk support, and improve exercise by passively stretching the hip flexors and lower back muscles (sacrospinalis) and aggressively strengthening the hamstring, gluteus maximus, and abdominal muscles. Disc herniation can happen when pressure is applied to the posterior portion of the lumbar vertebrae with extension. Logically, this is caused by greater lumbar lordosis; lumbar lordosis diminishes by reducing the force applied to the lumbar vertebra's posterior surface. By enhancing the vertebral disc's flexion and lowering disc herniation, the pressure would drop, and the likelihood of persistent lower back pain would get lower, which happens in William's flexion exercises. A balance between the stability of the extensor and flexor muscles of the spine can be built up [10]. The prevalent condition known as nonspecific low back pain (NSLBP) affects a sizable portion of the population. Its management often involves a multidisciplinary approach that includes a range of therapies, including medication, lifestyle modifications, and exercise. Because William's flexion exercises focus on lumbar spine flexibility and muscle strengthening, they have been considered one of the exercise modalities explored for NSLBP [11].

Maintaining strength requires doing core-strengthening workouts. Research indicates that for people with low back pain, core training significantly reduces pain levels. In contrast to core muscle training, strengthening the muscles in the back and lower limbs had less of an impact on pain relief. The phrase "nonspecific low back pain" is broad and encompasses a variety of conditions, with core strengthening being crucial [12]. Certain studies suggested general therapeutic exercise as a regular treatment for nonspecific low back pain to lessen the impairment caused by it [13]. The research by Bardin et al. [14] suggested self-management exercise in addition to hot packs as the initial course of treatment for NSLBP. Hot pack application is thought to be a pain management technique that relaxes muscles and has an analgesic effect. A healthcare guideline also suggests self-management exercise or an instructional home exercise program with hot packs for the treatment of low back pain. Exercise therapy in any form can help with pain management, increasing lumbar range of motion, enhancing functional activity, and raising quality of life [15]. William's flexion exercises were included because they were thought to improve lumbar flexibility and reduce pain by working on the muscles around the lower back. The purpose of selecting these exercises was to decrease stiffness, enhance mobility, and progressively increase tolerance to movement in the afflicted area. The most susceptible category is adults of working age since the frequency and incidence of LBP rise with age [16]. According to the comprehensive review of nonspecific low back pain by Gordon and Bloxham [17], increasing the flexibility of the back's muscles, tendons, and ligaments helps patients move more functionally and enhances their range of motion. Increasing the strength of core muscles can help stabilize the lumbar spine. According to Fatemi et al. [18], the results demonstrate that William's corrective training is a practical and effective technique for correcting and improving lumbar abnormalities.

## Conclusions

This study reveals the high frequency of nonspecific low back pain in healthy adults and indicates several risk factors, associated illnesses, and effects. The case report highlighted the positive impact of William's flexion exercises on NSLBP outcomes, showing enhanced range of motion and flexibility, decreased pain, heightened muscular strength, decreased disability, and enhanced quality of life. Medical schools can play a vital role in implementing these interventions to support normal individuals' physical and academic well-being. By doing so, they can help prevent and manage low back pain among normal individuals, thus improving their quality of life and performance.
